# Supplementary material for: Pandemic catch-22: The role of mobility restrictions and institutional inequalities in halting the spread of COVID-19
Source: PLoS One. 2021 Jun 28;16(6):e0253348. doi: 10.1371/journal.pone.0253348 (PMC8238194; doi:10.1371/journal.pone.0253348)

S1 Appendix to Pandemic Catch-22: The role of mobility restrictions and institutional inequalities in halting the spread of COVID-19

Adnan M. S. Fakir *   Tushar Bharati †

*Corresponding Author
University of Western Australia Business School, Economics Department, Perth, Australia.
BRAC University, Economics and Social Sciences, Dhaka, Bangladesh
email: adnan.fakir@uwa.edu.au; ORCID: 0000-0002-2291-2196

†University of Western Australia Business School, Economics Department, Perth, Australia.
email: tushar.bharati@uwa.edu.au; ORCID: 0000-0003-4115-9096

S1 Table: List of Countries

#	Country	Stringency Index	Cases Growth Rate (7-Day MA)
1	Afghanistan	66.18	0.10
2	Angola	78.01	0.06
3	Argentina*ψ	85.41	0.10
4	Aruba	53.29	0.04
5	Australia*ψ	56.47	0.05
6	Austria*ψ	54.49	0.07
7	Bahrain*ψ	67.48	0.07
8	Bangladesh*	77.74	0.09
9	Barbados	72.62	0.05
10	Belarus*ψ	11.86	0.09
11	Belgium*ψ	57.15	0.09
12	Belize	70.88	0.04
13	Benin	52.21	0.07
14	Bolivia*	89.80	0.08
15	Bosnia and Herzegovina	68.97	0.07
16	Botswana	69.43	0.05
17	Brazil*	69.49	0.11
18	Bulgaria*ψ	54.36	0.07
19	Burkina Faso	59.45	0.06
20	Cambodia	41.88	0.04
21	Cameroon	57.95	0.08
22	Canada*	58.47	0.06
23	Cape Verde	76.63	0.06
24	Chile*ψ	70.47	0.10
25	Colombia*ψ	80.89	0.11
26	Costa Rica*ψ	70.05	0.08
27	Cote d'Ivoire*ψ	64.77	0.09
28	Croatia*ψ	56.25	0.06
29	Czech Republic*ψ	51.85	0.07
30	Denmark*ψ	61.47	0.08
31	Dominican Republic	76.59	0.09
32	Ecuador*	80.21	0.08
33	Egypt	60.19	0.08
34	El Salvador*	92.61	0.08
35	Estonia*	45.82	0.06
36	Fiji*ψ	75.56	0.03
37	Finland*	42.49	0.06
38	France	62.60	0.07
39	Gabon	72.61	0.08
40	Georgia	75.56	0.08
41	Germany*ψ	53.35	0.07
42	Ghana*ψ	59.83	0.09
43	Greece*ψ	60.61	0.06
44	Guatemala	94.54	0.08
45	Haiti	74.17	0.08
46	Honduras	96.36	0.08
47	Hungary*ψ	61.80	0.06
48	India*ψ	71.40	0.09
49	Indonesia*	62.09	0.09
50	Iraq	86.47	0.08
51	Ireland*ψ	65.42	0.09
52	Israel*ψ	70.28	0.08
53	Italy*ψ	68.51	0.09
54	Jamaica	74.98	0.05
55	Japan*	35.36	0.04
56	Jordan	67.87	0.07
57	Kazakhstan*ψ	81.29	0.08
58	Kenya*ψ	86.81	0.08
59	Kuwait*ψ	80.22	0.07
60	Kyrgyzstan	82.17	0.08
61	Laos	55.92	0.02
62	Latvia*ψ	52.96	0.06
63	Lebanon	68.44	0.06
64	Libya	94.67	0.08
65	Lithuania*ψ	58.38	0.06
66	Luxembourg*ψ	49.18	0.08
67	Malaysia*ψ	56.62	0.04
68	Mali	60.32	0.07
69	Mauritius	59.76	0.04
70	Mexico*	64.70	0.09
71	Moldova	76.82	0.09
72	Mongolia	66.54	0.05
73	Morocco*	77.07	0.08
74	Mozambique	63.77	0.08
75	Myanmar*	79.04	0.05
76	Namibia	61.99	0.06
77	Nepal*ψ	77.61	0.06
78	Netherlands*	58.95	0.08
79	New Zealand*ψ	48.32	0.06
80	Nicaragua	12.06	0.08
81	Niger	39.38	0.07
82	Nigeria*ψ	67.09	0.08
83	Norway*ψ	49.80	0.07
84	Oman	78.16	0.08
85	Pakistan*ψ	72.36	0.09
86	Panama*	82.73	0.08
87	Papua New Guinea	58.14	0.04
88	Paraguay*ψ	83.72	0.07
89	Peru*ψ	85.91	0.10
90	Philippines*ψ	78.48	0.07
91	Poland*ψ	61.27	0.09
92	Portugal*ψ	69.63	0.08
93	Puerto Rico	83.36	0.05
94	Qatar*ψ	75.62	0.10
95	Romania*	60.96	0.08
96	Russia*ψ	65.27	0.09
97	Rwanda*ψ	72.63	0.06
98	Saudi Arabia*ψ	76.57	0.11
99	Senegal*ψ	58.47	0.07
100	Serbia*ψ	72.64	0.13
101	Singapore*ψ	59.15	0.04
102	Slovakia*ψ	59.48	0.06
103	Slovenia*ψ	53.88	0.05
104	South Africa*ψ	75.77	0.11
105	South Korea*ψ	55.14	0.04
106	Spain*	59.29	0.09
107	Sri Lanka	62.81	0.05
108	Sweden	34.62	0.08
109	Switzerland*	53.74	0.08
110	Taiwan*ψ	23.90	0.02
111	Tajikistan	60.97	0.10
112	Tanzania	37.94	0.06
113	Thailand*ψ	54.79	0.03
114	Togo*ψ	59.86	0.06
115	Trinidad and Tobago	73.68	0.05
116	Turkey*ψ	67.31	0.12
117	Uganda	85.33	0.06
118	Ukraine*ψ	68.90	0.09
119	United Arab Emirates*ψ	60.43	0.06
120	United Kingdom*ψ	58.32	0.07
121	United States*	60.49	0.09
122	Uruguay*	53.00	0.05
123	Venezuela	83.12	0.06
124	Vietnam*ψ	62.37	0.02
125	Yemen	54.66	0.08
126	Zambia	48.44	0.07
127	Zimbabwe*	79.31	0.07

Mean of stringency index & cases 7-day moving average growth rate values from 15 Feb to 30 July, 2020.
* denotes countries with available testing data for a duration of the time frame.
ψ denotes countries who reported to conduct full contact tracing sometime within the time frame.

S2 Table: Data Sources

Variable	Data Source
Stringency Index	Oxford Government Response Tracker
Retail and Recreation (% change)	Google Community Mobility Report
Grocery and Pharmacy (% change)	Google Community Mobility Report
Parks (% change)	Google Community Mobility Report
Transit Stations (% change)	Google Community Mobility Report
Workplaces (% change)	Google Community Mobility Report
Residential (% change)	Google Community Mobility Report
Tests (Growth Rate)	Our World In Data (OWID)
Confirmed Cases (Growth Rate)	Johns Hopkins Center for Systems Science and Engineering (CSSE)
Deaths (Growth Rate)	Johns Hopkins Center for Systems Science and Engineering (CSSE)
Recoveries (Growth Rate)	Johns Hopkins Center for Systems Science and Engineering (CSSE)
Population Density	World Development Indicators (most recent year available)
Primary Education	World Development Indicators (most recent year available)
Poverty Headcount (2011 PPP)	World Development Indicators (most recent year available)
Gini Index	World Development Indicators (most recent year available)
Population Aged 65 or older	World Development Indicators (most recent year available)
PM2.5 (2010-2017 Average)	World Development Indicators
Hospital Beds per 100k Population	OECD, Eurostat, World Bank, National Government Records, and other sources (most recent year available since 2010)
Handwashing Facilities	United Nations Statistics Division (most recent year available)
CVD Death Rate	Global Burden of Disease Study 2017 Results
Democracy Score	Economist Intelligence Unit (EIU)
Governance Effectiveness	Worldwide Governance Indicators (WGI)
Corruption Perception Index	Transparency International (TI)

S3 Table: Summary statistics by level of development

	N	Mean	SD	Median	Min	Max	N	Mean	SD	Median	Min	Max
	Developing Countries						Developed Countries					
Oxford Government Response Tracker												
Stringency Index	6209	65.45	24.22	70.83	0.00	100.00	12113	64.31	24.24	71.30	0.00	100.00
Google Mobility Measures												
Retail and Recreation (% change)	6209	-29.94	24.78	-25.00	-94.00	35.00	12113	-35.09	27.23	-33.00	-97.00	42.00
Grocery and Pharmacy (% change)	6209	-15.97	22.13	-13.00	-95.00	94.00	12108	-14.61	22.50	-10.00	-97.00	84.00
Parks (% change)	6208	-18.43	23.51	-16.00	-90.00	141.00	12113	-1.72	62.64	-15.00	-95.00	517.00
Transit Stations (% change)	6209	-34.15	23.74	-32.00	-92.00	39.00	12113	-40.57	24.65	-43.00	-95.00	27.00
Workplaces (% change)	6209	-21.24	22.25	-18.00	-87.00	67.00	12113	-30.28	22.47	-29.00	-92.00	80.00
Residential (% change)	6165	12.38	9.32	11.00	-16.00	49.00	12081	13.54	10.54	13.00	-14.00	55.00
Outbreak Variables (Growth Rates of 7-Day Moving Average per million population)												
Tests	2673	0.05	0.06	0.03	0.00	0.97	7987	0.05	0.06	0.02	-0.01	0.70
Cases	6209	0.07	0.12	0.03	-0.13	1.67	12113	0.07	0.14	0.02	-0.01	2.65
Cases to Tests	2673	0.00	0.05	0.00	-0.49	0.61	7987	0.01	0.06	-0.01	-0.34	1.53
Deaths	4714	0.07	0.15	0.03	0.00	2.03	10555	0.07	0.18	0.02	-0.01	4.01
Deaths to Tests	2305	0.01	0.09	-0.00	-0.44	1.80	7371	0.02	0.11	-0.00	-0.24	3.24
Days since first case (by Country)	6209	72.69	43.04	71.00	-76.00	188.00	12113	79.20	45.52	78.00	-46.00	194.00
Heterogeneity Variables												
Population Density	6045	157.31	210.56	82.81	1.98	1265.04	12113	269.40	931.61	88.13	3.08	7915.73
Primary Education	3236	50.20	22.58	48.83	13.87	99.44	8321	89.90	10.50	92.70	61.80	100.00
Poverty Headcount (2011 PPP)	5772	23.18	20.17	18.80	0.00	62.90	10522	1.88	3.66	0.50	0.00	18.90
Gini Index	5772	39.63	7.11	40.30	25.70	57.10	10380	36.89	8.19	34.90	24.20	63.00
Population Aged 65 or older	6045	4.60	2.58	4.03	2.17	16.46	12113	12.72	6.40	13.93	1.14	27.05
PM2.5 (2010-2017 Average)	5520	40.43	21.49	33.11	13.80	98.25	11699	22.50	16.23	17.81	6.46	87.02
Corruption Perception Index	5919	32.12	8.79	32.00	14.00	57.00	11454	54.83	19.32	53.00	17.00	88.00
Democracy Score	5816	4.71	1.43	4.92	1.93	7.73	11596	6.66	2.06	7.16	1.93	9.87
Governance Effectiveness	6083	-0.54	0.58	-0.58	-2.24	1.36	12113	0.59	0.88	0.49	-1.85	2.23
Hospital Beds per 100k Population	5217	1.64	1.93	0.90	0.10	8.80	11719	3.85	2.58	2.97	0.60	13.05
Handwashing Facilities	5363	45.62	27.91	41.95	2.73	90.65	2669	80.31	16.99	87.85	43.99	99.00
CVD Death Rate	6209	307.43	116.12	276.05	103.96	597.03	11976	200.22	100.04	171.29	79.37	496.22

Low- and lower middle-income countries are categorized as developing countries while high- and upper middle-income countries are categorized as developed countries. Low-income economies are defined as those with a GNI per capita, calculated using the World Bank Atlas method, of $1,025 or less in 2018; lower middle-income economies are those with a GNI per capita between $1,026 and $3,995; upper middle-income economies are those with a GNI per capita between $3,996 and $12,375; high-income economies are those with a GNI per capita of $12,376 or more.

S4 Table: Mean of 7-day moving average growth rates by testing approach

**Testing Approach**	**Tests**	**Cases**	**Cases:Tests**	**Deaths**	**Deaths:Tests**
Full Sample	0.0467	0.0572	0.0061	0.0610	0.0179
Contact Tracing					
No Contract Tracing	0.0867	0.1185	0.0196	0.1125	0.0410
Limited Contract Tracing	0.0470	0.0600	0.0083	0.0653	0.0179
Full Contract Tracing	0.0410	0.0467	0.0029	0.0524	0.0152
Testing Policy					
No Testing Policy	0.1185	0.1999	0.0601	0.2562	0.1366
Limited Testing	0.0670	0.0874	0.0137	0.1003	0.0414
Symptom Testing	0.0376	0.0400	0.0005	0.0412	0.0036
Open Testing	0.0279	0.0283	0.0003	0.0326	0.0038
Testing Report					
People Tested	0.0449	0.0571	0.0091	0.0576	0.0148
Total Tests	0.0476	0.0585	0.0055	0.0621	0.0195

Using available data from 15 Feb to 30 July, 2020. Each row within each panel represents the corresponding restricted sample. *Limited contact tracing* is when tracing is not done for all cases; *Full contact tracing* is when tracing is done for all identified cases; *Limited testing policy* tests only those who both (a) have symptoms AND (b) meet specific criteria (eg. key workers, admitted to hospital, came into contact with a known case, returned from overseas); *Symptom testing policy* tests anyone showing Covid-19 symptoms; *Open testing policy* is when testing is open to all public (eg. ”drive through” testing available to asymptomatic people); *People tested* refers to testing data where the number of people tested (excluding follow-up or repeated tests of the same person) are reported; *Totals tests* is when only the total number of tests conducted is reported, regardless of double counting.

S5 Table: Impact on mobility using alternative instruments

VARIABLES	(1) Retail Recreation	(2) Grocery Pharmacy	(3) Parks	(4) Transit Stations	(5) Workplaces	(6) Residential
2SLS: World excluding Country IV						
Stringency Index (Lag 0)	-1.12***	-0.69***	-0.63***	-1.05***	-0.92***	0.38***
	(0.05)	(0.04)	(0.09)	(0.04)	(0.04)	(0.02)
Mean of DV	-33.34	-15.07	-7.38	-38.39	-27.22	13.14
F-Stat	289.42	289.37	289.43	289.42	289.42	289.19
Stringency Index (Lag 14)	-0.67***	-0.47***	-0.29***	-0.62***	-0.56***	0.24***
	(0.04)	(0.03)	(0.09)	(0.04)	(0.04)	(0.02)
Mean of DV	-34.17	-15.50	-7.73	-39.31	-27.88	13.45
F-Stat	318.47	318.32	318.48	318.47	318.47	317.12
2SLS: Region excluding Country IV						
Stringency Index (Lag 0)	-1.13***	-0.62***	-0.96***	-1.03***	-0.83***	0.38***
	(0.05)	(0.04)	(0.11)	(0.04)	(0.04)	(0.02)
Mean of DV	-33.34	-15.07	-7.38	-38.39	-27.22	13.14
F-Stat	304.39	304.21	304.39	304.39	304.39	303.18
Stringency Index (Lag 14)	-0.65***	-0.42***	-0.49***	-0.58***	-0.48***	0.22***
	(0.05)	(0.04)	(0.08)	(0.05)	(0.04)	(0.02)
Mean of DV	-34.17	-15.50	-7.73	-39.31	-27.88	13.45
F-Stat	312.02	311.85	312.01	312.02	312.02	310.14
2SLS: World excluding Region IV						
Stringency Index (Lag 0)	-1.11***	-0.71***	-0.56***	-1.05***	-0.94***	0.38***
	(0.05)	(0.04)	(0.10)	(0.04)	(0.04)	(0.02)
Mean of DV	-33.34	-15.07	-7.38	-38.39	-27.22	13.14
F-Stat	245.36	245.32	245.37	245.36	245.36	245.03
Stringency Index (Lag 14)	-0.67***	-0.48***	-0.25***	-0.63***	-0.58***	0.24***
	(0.05)	(0.04)	(0.09)	(0.04)	(0.04)	(0.02)
Mean of DV	-34.17	-15.50	-7.73	-39.31	-27.88	13.45
F-Stat	267.70	267.59	267.71	267.70	267.70	266.40
2SLS: Sub-region excluding Country IV						
Stringency Index (Lag 0)	-1.11***	-0.66***	-1.00***	-1.01***	-0.83***	0.39***
	(0.05)	(0.04)	(0.10)	(0.04)	(0.04)	(0.02)
Mean of DV	-33.34	-15.07	-7.38	-38.39	-27.22	13.14
F-Stat	237.41	237.39	237.41	237.41	237.41	237.66
Stringency Index (Lag 14)	-0.72***	-0.48***	-0.74***	-0.64***	-0.52***	0.26***
	(0.05)	(0.04)	(0.09)	(0.05)	(0.05)	(0.02)
Mean of DV	-34.17	-15.50	-7.73	-39.31	-27.88	13.45
F-Stat	234.29	234.35	234.29	234.29	234.29	233.84
Observations (Lag 0)	18,324	18,319	18,323	18,324	18,324	18,248
Observations (Lag 14)	17,852	17,847	17,851	17,852	17,852	17,776
Number of country	127	127	127	127	127	127
Fixed Effects	Country; Days since first case					

Robust standard errors clustered at the country level.
* $p < 0.10$; ** $p < 0.05$; *** $p < 0.01$.

S6 Table: Impact on growth rates using alternative instruments

VARIABLES	(1) Tests	(2) Cases	(3) Cases:Tests	(4) Deaths	(5) Deaths:Tests
2SLS: World excluding Country IV					
Stringency Index (Lag 0)	-0.0007**	-0.0015***	-0.0010**	-0.0059***	-0.0049***
	(0.0003)	(0.0003)	(0.0005)	(0.0013)	(0.0018)
Mean of DV	0.047	0.071	0.006	0.070	0.018
F-Stat	46.409	289.425	46.409	70.597	32.534
Stringency Index (Lag 14)	-0.0008***	-0.0023***	-0.0010***	-0.0046***	-0.0031***
	(0.0002)	(0.0002)	(0.0002)	(0.0005)	(0.0006)
Mean of DV	0.046	0.069	0.006	0.069	0.017
F-Stat	86.301	318.467	86.301	202.627	59.742
2SLS: Region excluding Country IV					
Stringency Index (Lag 0)	-0.0005**	-0.0015***	-0.0008**	-0.0044***	-0.0015
	(0.0002)	(0.0003)	(0.0003)	(0.0010)	(0.0010)
Mean of DV	0.047	0.071	0.006	0.070	0.018
F-Stat	60.606	304.391	60.606	114.018	37.607
Stringency Index (Lag 14)	-0.0009***	-0.0024***	-0.0010***	-0.0041***	-0.0025***
	(0.0002)	(0.0002)	(0.0002)	(0.0004)	(0.0006)
Mean of DV	0.046	0.069	0.006	0.069	0.017
F-Stat	96.588	312.025	96.588	293.061	94.064
2SLS: World excluding Region IV					
Stringency Index (Lag 0)	-0.0007**	-0.0015***	-0.0011**	-0.0063***	-0.0059**
	(0.0003)	(0.0003)	(0.0005)	(0.0014)	(0.0023)
Mean of DV	0.047	0.071	0.006	0.070	0.018
F-Stat	38.694	245.362	38.694	59.111	26.549
Stringency Index (Lag 14)	-0.0008***	-0.0022***	-0.0011***	-0.0047***	-0.0033***
	(0.0002)	(0.0002)	(0.0002)	(0.0005)	(0.0006)
Mean of DV	0.046	0.069	0.006	0.069	0.017
F-Stat	72.765	267.696	72.765	165.143	49.611
2SLS: Sub-region excluding Country IV					
Stringency Index (Lag 0)	-0.0003	-0.0009***	-0.0004	-0.0042***	-0.0024**
	(0.0002)	(0.0003)	(0.0004)	(0.0010)	(0.0010)
Mean of DV	0.047	0.071	0.006	0.070	0.018
F-Stat	86.176	237.407	86.176	93.581	36.018
Stringency Index (Lag 14)	-0.0006***	-0.0017***	-0.0005**	-0.0041***	-0.0024***
	(0.0002)	(0.0003)	(0.0002)	(0.0005)	(0.0005)
Mean of DV	0.046	0.069	0.006	0.069	0.017
F-Stat	97.907	234.292	97.907	176.617	77.756
Observations (Lag 0)	10,662	18,324	10,662	15,270	9,678
Observations (Lag 14)	10,539	17,852	10,539	15,151	9,625
Number of country	80	127	80	121	78
Fixed Effects	Country; Days since first case				

Robust standard errors clustered at the country level.
* $p < 0.10$; ** $p < 0.05$; *** $p < 0.01$.

S7 Table: Impact on 7-day moving average growth rates by testing approach

VARIABLES	(1) Full Sample	(2) No Testing Policy	(3) Limited Testing	(4) Symptom Testing	(5) Open Testing	(6) No Contact Tracing	(7) Limited Contact Tracing	(8) Full Contact Tracing	(9) People Tested	(10) Total Tests
Tests										
Stringency Index	-0.0009***	-0.0002	-0.0005	-0.0015***	-0.0018***	-0.0009	-0.0014***	-0.0005**	-0.0007***	-0.0008**
(Lag 14)	(0.0002)	(0.0013)	(0.0004)	(0.0005)	(0.0006)	(0.0007)	(0.0003)	(0.0003)	(0.0002)	(0.0003)
Observations	10,539	263	3,527	3,664	2,866	933	3,572	5,815	2,832	6,198
Number of country	80	12	53	51	34	20	40	59	21	48
Mean of DV	0.046	0.118	0.066	0.037	0.027	0.086	0.047	0.040	0.044	0.047
F-Stat	78.483	3.652	32.985	26.782	8.532	34.097	45.983	35.720	13.042	59.329
Cases:Tests										
Stringency Index	-0.0011***	0.0007	-0.0010**	-0.0014***	-0.0010***	-0.0016***	-0.0011***	-0.0009***	-0.0012***	-0.0011***
(Lag 14)	(0.0002)	(0.0008)	(0.0004)	(0.0004)	(0.0003)	(0.0003)	(0.0002)	(0.0003)	(0.0003)	(0.0004)
Observations	10,539	263	3,527	3,664	2,866	933	3,572	5,815	2,832	6,198
Number of country	80	12	53	51	34	20	40	59	21	48
Mean of DV	0.006	0.069	0.014	-0.001	-0.000	0.019	0.008	0.003	0.009	0.005
F-Stat	78.483	3.652	32.985	26.782	8.532	34.097	45.983	35.720	13.042	59.329
Deaths:Tests										
Stringency Index	-0.0032***	-0.0238	-0.0032***	-0.0028***	-0.0016***	-0.0022	-0.0025***	-0.0030***	-0.0015**	-0.0046***
(Lag 14)	(0.0006)	(0.0152)	(0.0009)	(0.0009)	(0.0004)	(0.0019)	(0.0007)	(0.0008)	(0.0006)	(0.0009)
Observations	9,625	159	3,080	3,563	2,612	768	3,451	5,196	2,709	5,639
Number of country	78	7	53	49	32	16	40	57	21	47
Mean of DV	0.017	0.137	0.041	0.004	0.002	0.041	0.018	0.014	0.013	0.019
F-Stat	55.454	1.404	24.276	24.206	6.919	27.599	37.831	21.820	11.925	45.380
Fixed Effects	Country; Days since first case									

Robust standard errors clustered at the country level.
* $p < 0.10$; ** $p < 0.05$; *** $p < 0.01$.

S8 Table: Heterogenous impact of transit station mobility on growth rates: Recursive mixed-process model (Full contract tracing sample)

VARIABLES	(1)<br>Cases:Tests	(2)<br>Deaths:Tests	(3)<br>Cases:Tests	(4)<br>Deaths:Tests	(5)<br>Cases:Tests	(6)<br>Deaths:Tests	(7)<br>Cases:Tests	(8)<br>Deaths:Tests
	< Median		> Median		< Median		> Median	
	Population Density				Corruption Perception Index			
Transit Stations	0.0029**	0.0111***	0.0010	0.0039***	-0.0003	0.0038	0.0015	0.0041***
	(0.0011)	(0.0032)	(0.0015)	(0.0014)	(0.0012)	(0.0043)	(0.0011)	(0.0015)
Observations	3,807	5,246	5,246	5,203	3,149	3,135	5,904	5,875
Mean of DV	0.007	0.016	0.002	0.015	0.009	0.015	0.001	0.015
	Primary Education				Democracy Score			
Transit Stations	-0.0004	0.0019**	0.0012	0.0053***	-0.0003	0.0060***	0.0025***	0.0064***
	(0.0011)	(0.0008)	(0.0010)	(0.0014)	(0.0007)	(0.0025)	(0.0009)	(0.0021)
Observations	2,207	2,193	2,193	6,846	3,970	3,942	5,083	5,068
Mean of DV	0.003	0.015	0.003	0.015	0.006	0.020	0.001	0.013
	Poverty Head Count				Government Effectiveness			
Transit Stations	0.0026	0.0057***	-0.0001	0.0020	-0.0002	0.0037**	0.0012	0.0076***
	(0.0024)	(0.0016)	(0.0007)	(0.0013)	(0.0013)	(0.0017)	(0.0011)	(0.0027)
Observations	4,283	4,254	4,254	4,770	3,764	5,275	5,275	5,246
Mean of DV	0.001	0.015	0.005	0.015	0.007	0.013	0.001	0.016
	Gini Index				Hospital Beds per 100k			
Transit Stations	0.0017***	0.0048***	0.0015	0.0042***	-0.0000	0.0044***	0.0024***	0.0057***
	(0.0005)	(0.0021)	(0.0012)	(0.0016)	(0.0005)	(0.0014)	(0.0008)	(0.0018)
Observations	3,843	3,811	5,210	5,199	3,425	3,386	5,628	5,624
Mean of DV	0.001	0.017	0.005	0.014	0.004	0.013	0.002	0.016
	Age 65 & Older				Handwashing Facilities			
Transit Stations	-0.0000	0.0046***	0.0025***	0.0047***	-0.0003	0.0032**	0.0012**	0.0046***
	(0.0005)	(0.0012)	(0.0009)	(0.0021)	(0.0006)	(0.0016)	(0.0010)	(0.0013)
Observations	4,240	4,201	4,201	4,813	1,976	1,962	7,077	7,048
Mean of DV	0.006	0.019	0.001	0.013	0.008	0.021	0.002	0.014
	PM2.5				Cardiovascular Diseases Death Rate			
Transit Stations	0.0023***	0.0047***	-0.0006	0.0057***	0.0028***	0.0068***	-0.0000	0.0038
	(0.0006)	(0.0016)	(0.0008)	(0.0022)	(0.0014)	(0.0021)	(0.0007)	(0.0032)
Observations	4,056	4,045	4,045	4,997	4,112	4,108	4,941	4,902
Mean of DV	0.001	0.014	0.005	0.016	0.002	0.014	0.004	0.016
Fixed Effects	Country; Days since first case							

Robust standard errors clustered at the country level.
* $p < 0.10$; ** $p < 0.05$; *** $p < 0.01$.

S1 Figure: Event graph of OxCGRT Stringency Index by country over time

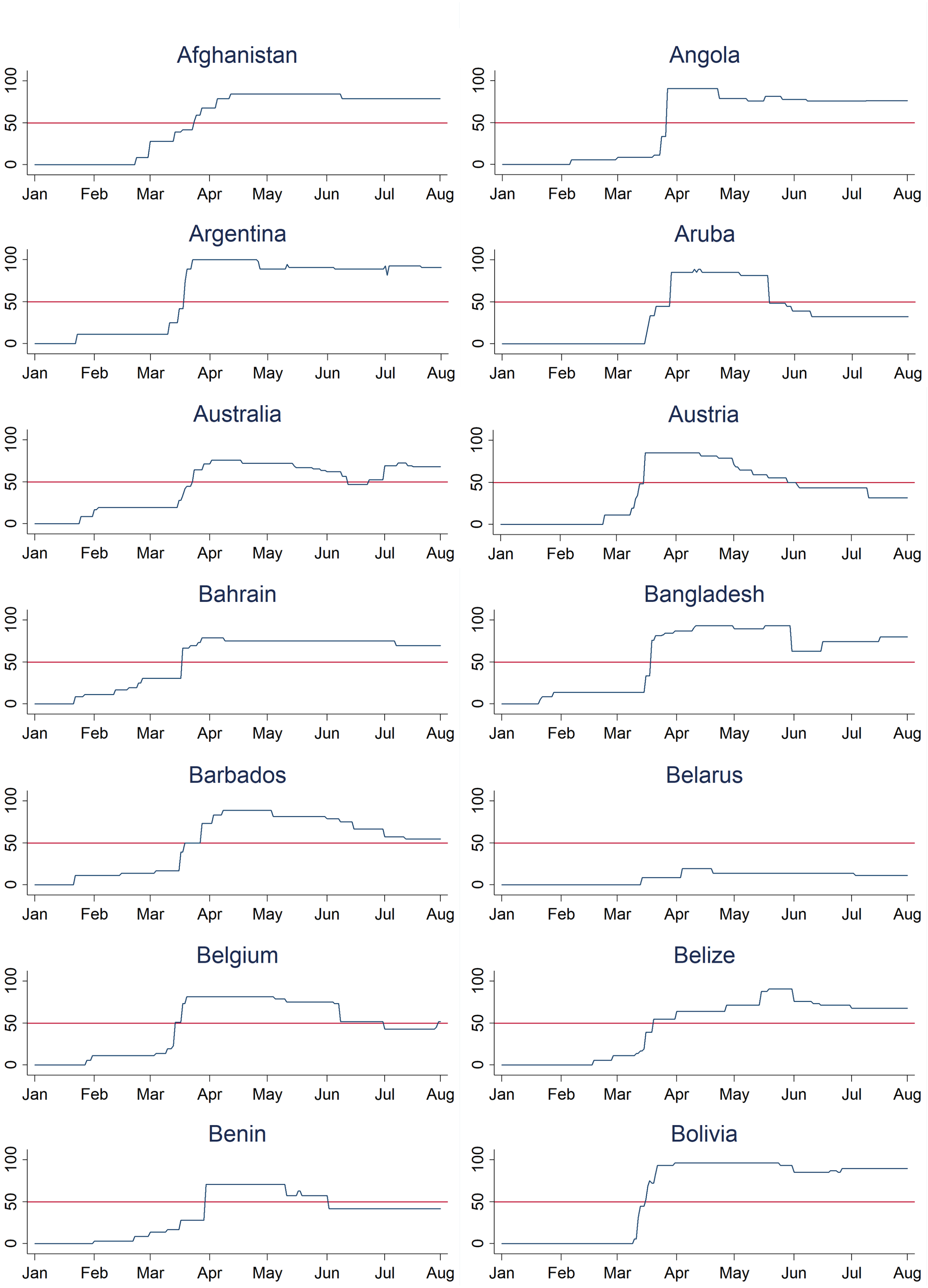

S2 Figure: Event graph of OxCGRT Stringency Index by country over time (cont.)

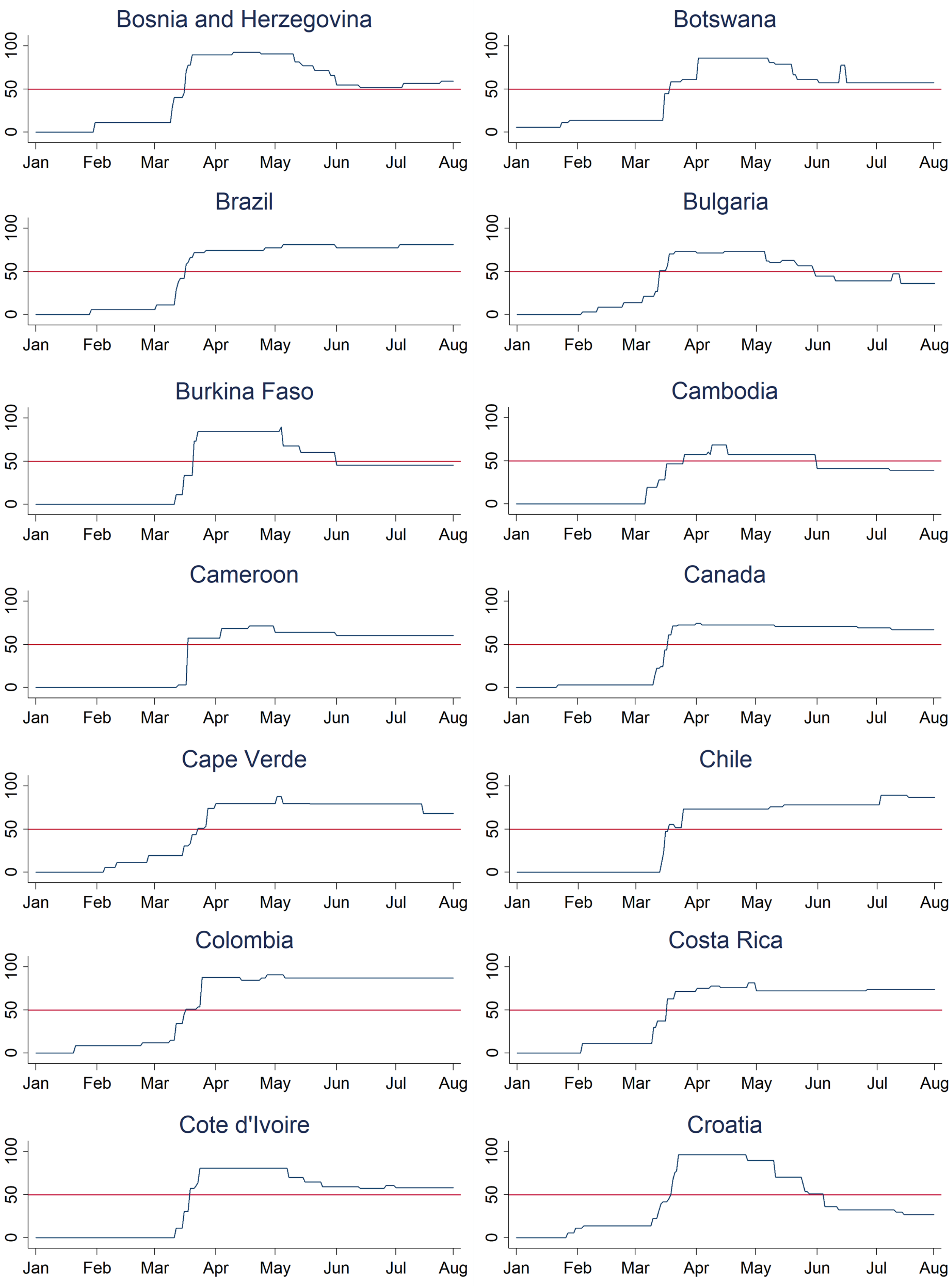

S3 Figure: Event graph of OxCGRT Stringency Index by country over time (cont.)

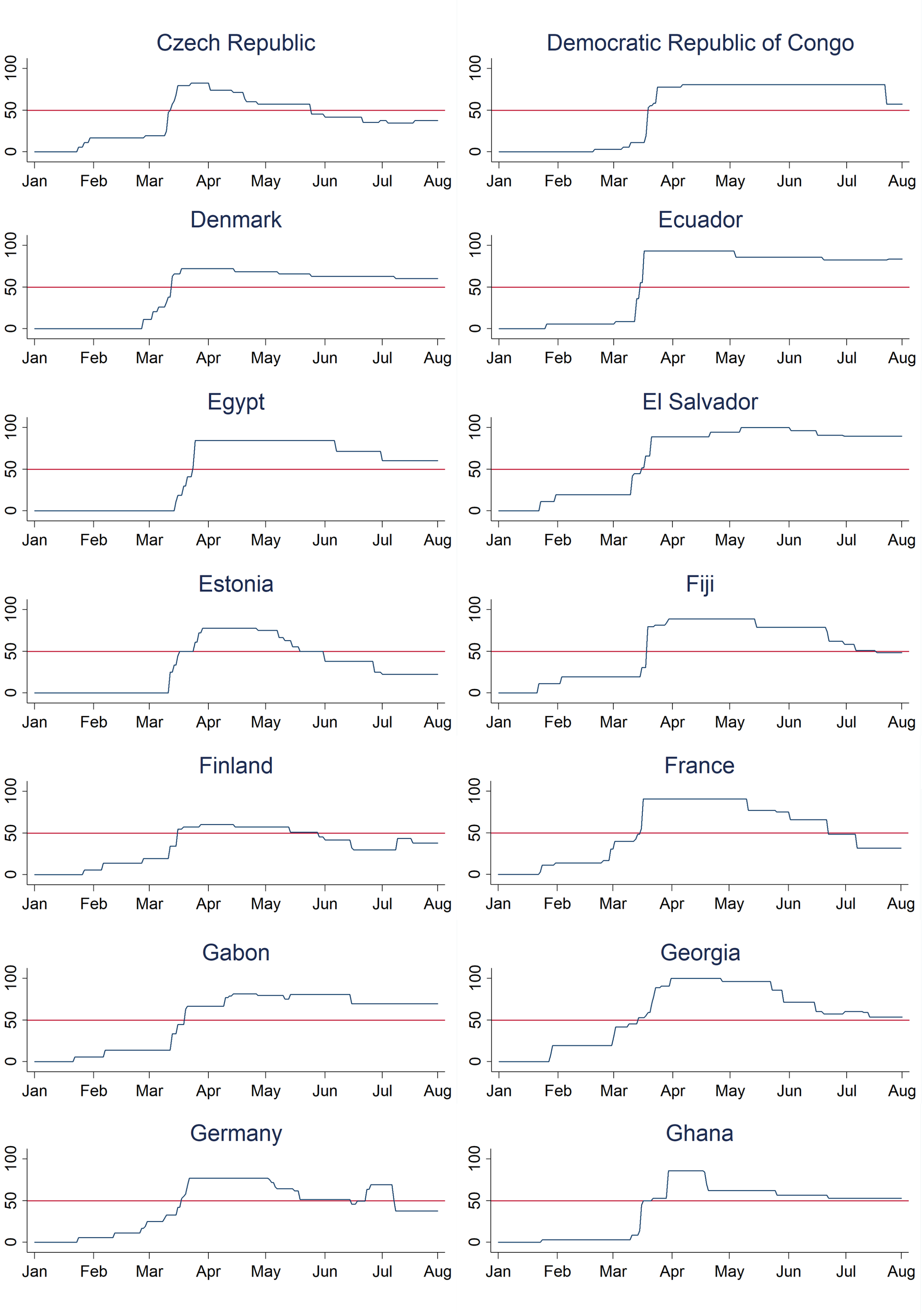

S4 Figure: Event graph of OxCGRT Stringency Index by country over time (cont.)

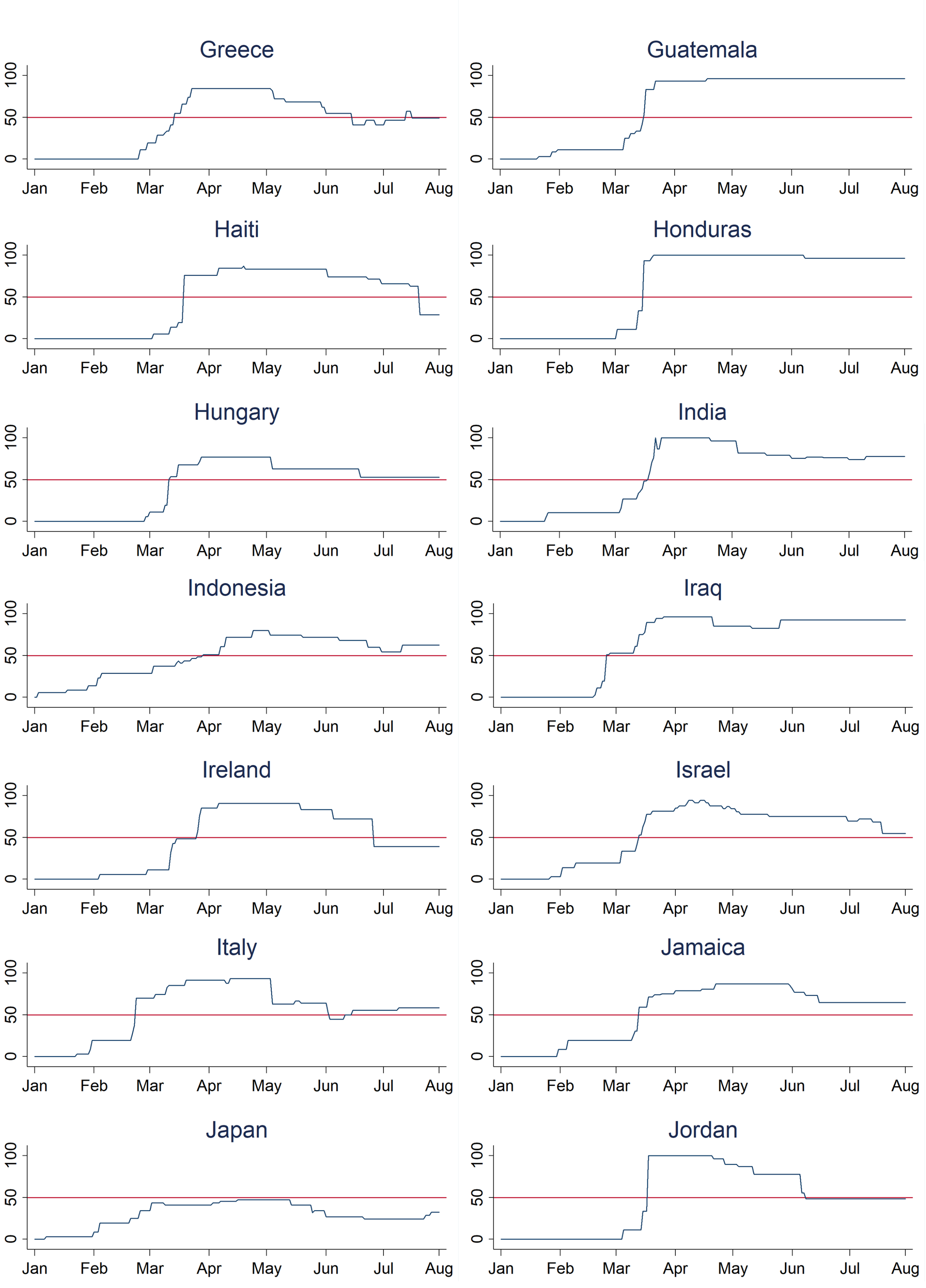

S5 Figure: Event graph of OxCGRT Stringency Index by country over time (cont.)

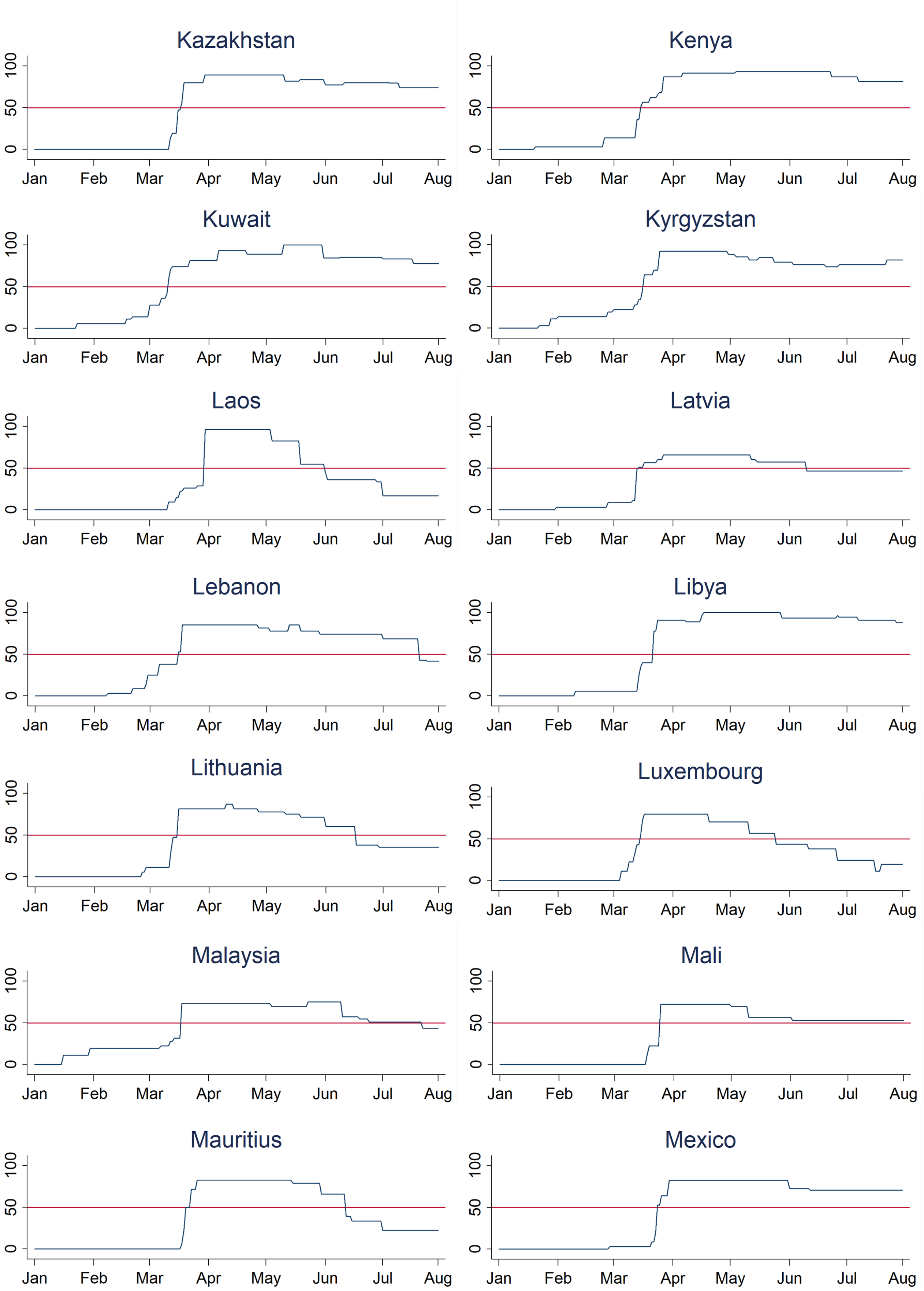

S6 Figure: Event graph of OxCGRT Stringency Index by country over time (cont.)

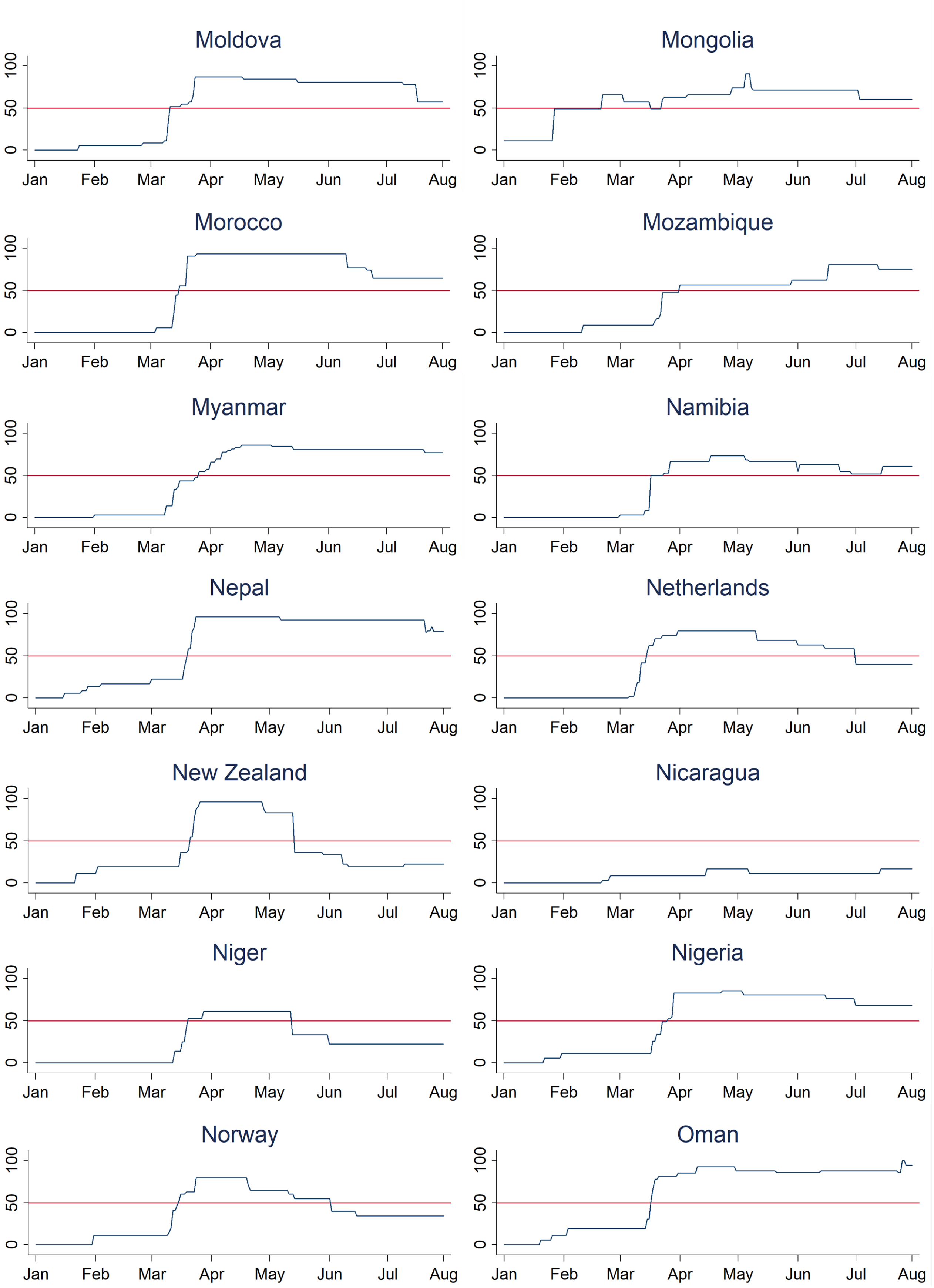

S7 Figure: Event graph of OxCGRT Stringency Index by country over time (cont.)

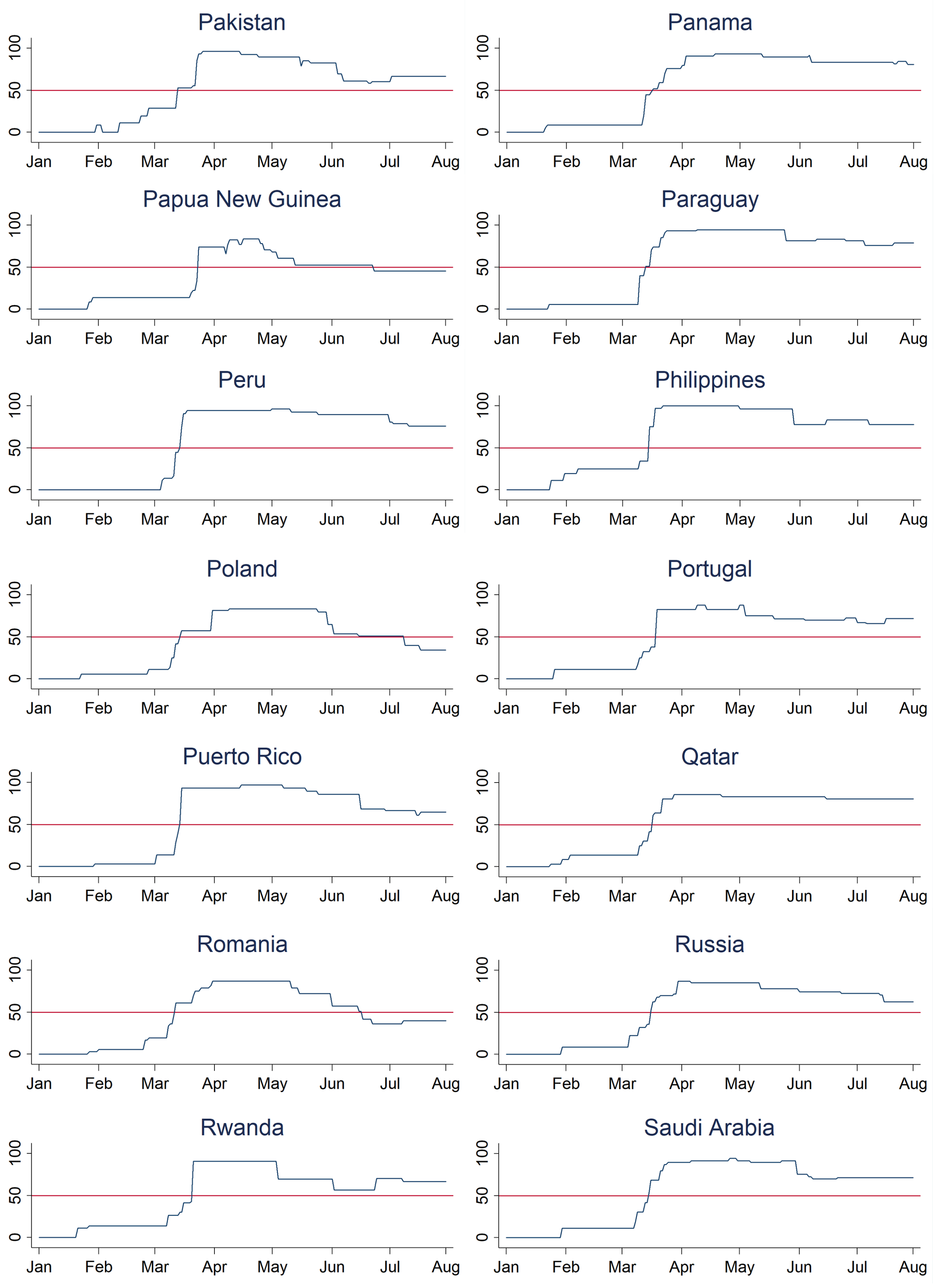

S8 Figure: Event graph of OxCGRT Stringency Index by country over time (cont.)

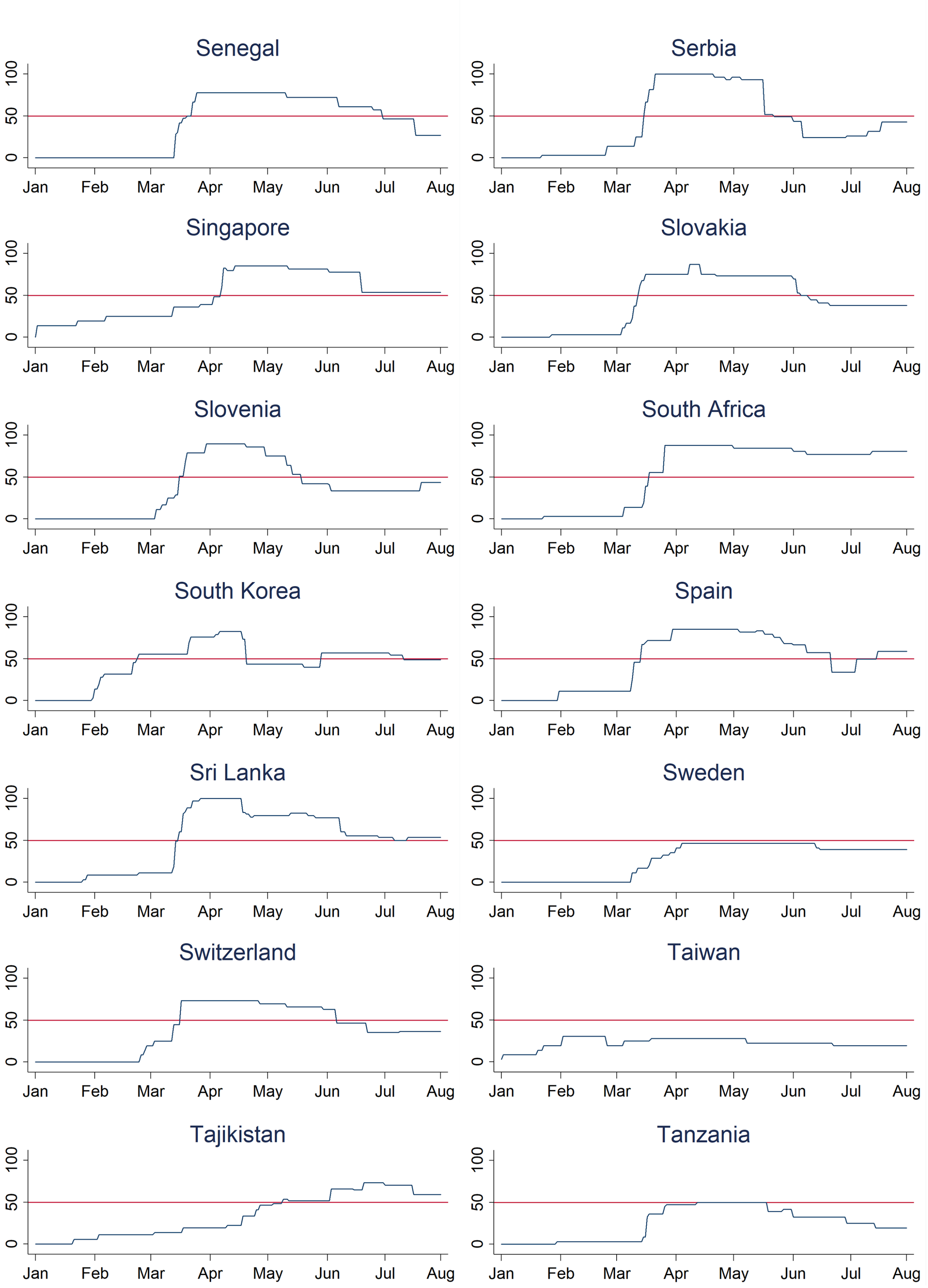

S9 Figure: Event graph of OxCGRT Stringency Index by country over time (cont.)

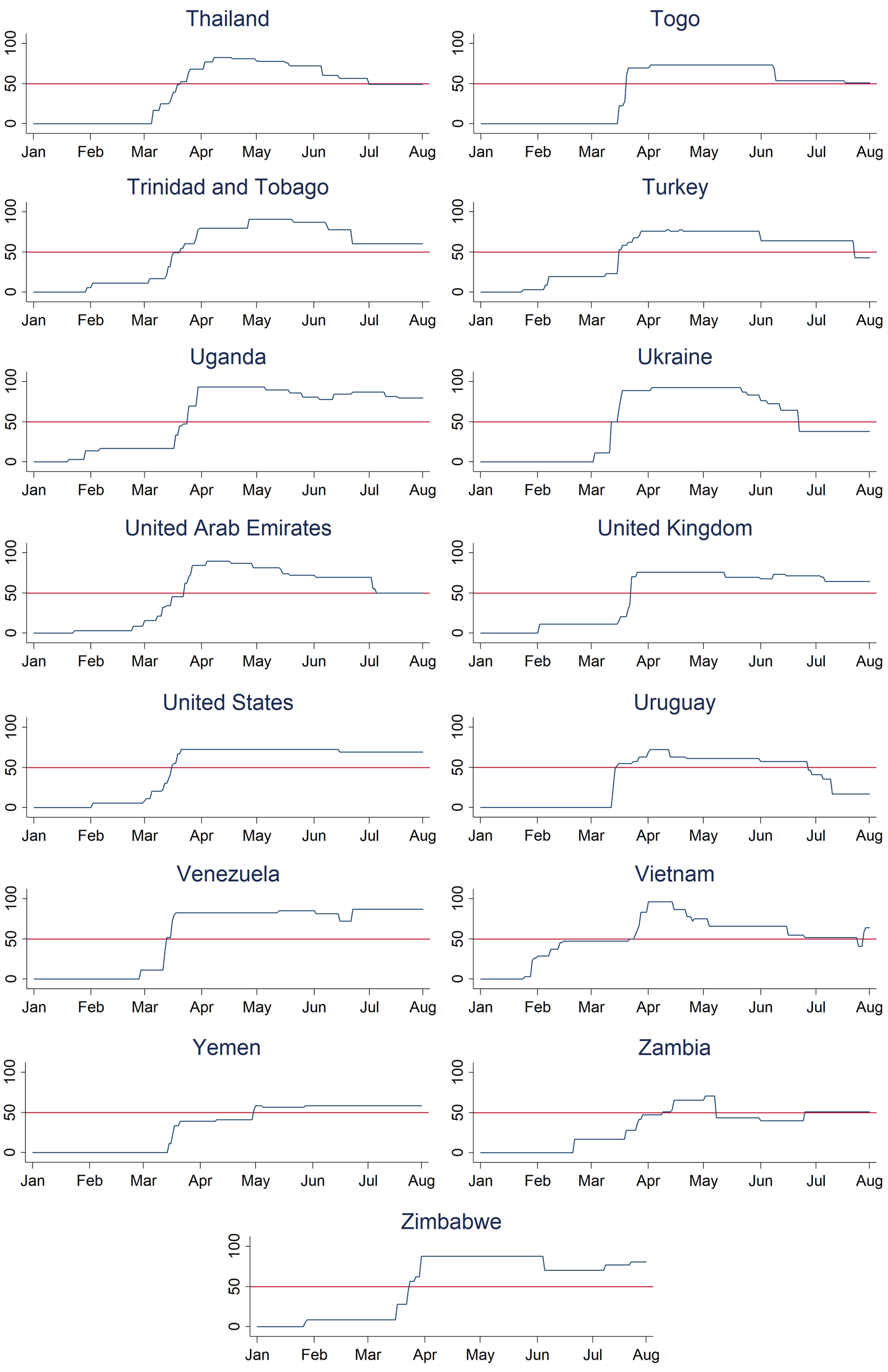

Supplement: S1 Appendix — (PDF) [file pone.0253348.s001.pdf]
