# Supplementary material for: Pandemic catch-22: The role of mobility restrictions and institutional inequalities in halting the spread of COVID-19
Source: PLoS One. 2021 Jun 28;16(6):e0253348. doi: 10.1371/journal.pone.0253348 (PMC8238194; doi:10.1371/journal.pone.0253348)
Supplement: Attachment — Submitted filename: Response to Reviewers.pdf [file pone.0253348.s002.pdf]

Response to reviewers

Response to reviewer 1:

Reviewer #1: This is an original study which aims to estimate the causal effect of mobility and activity restrictions, and the growth rate of confirmed cases and deaths attributed to COVID-19. Overall, this is a well-designed, implemented, and written manuscript.

The authors did not describe the mobility and activity restriction characteristics according to country. This is mandatory because several countries adopted a rigid restrictions, while other countries adopted a flexible restrictions.

Authors: Thank you for raising this pertinent point. We discuss this in section 2.1.2 when describing the Oxford Stringency Index. We agree that the variation in the stringency measures needs to be carefully addressed and made transparent. In response to the reviewer's comment, we have now included a set of event graphs that report the variation in the Stringency Index by country over time. These graphs, one for each of the 127 countries in our sample, are collected under Figures S1 to S9 in the appendix (Supporting Information). We have also incorporated the following paragraph in section 2.1.2.

"It is also important to note while some countries enacted rigid mobility and activity restrictions, other countries adopted more flexible measures. Further, these levels of flexibility/rigidity have changed within a country over time. OxCGRT integrates these fluctuations into their stringency index by categorizing each of the nine indicators into ordinal levels by the rigidity of the restriction. For example, school closures are categorized into ``0 - no measures; 1 - recommend closing or all schools open with alterations resulting in significant differences compared to non-Covid-19 operations; 2 - require closing (only some levels or categories, for eg. just high school, or just public schools); 3 - require closing all levels'' (Hale and Webster, 2020). The final stringency index is then a composite weighted index where higher values reflect the levels of rigidity of the restrictions. Please refer to Hale and Webster (2020) for details on the index's construction. Figures S1 to S9 in the appendix provide event graphs of the stringency index by country over time for all 127 countries in our sample. Values above 50 can be interpreted as the country undertaking relatively stricter measures."

Reviewer #1: I recommend to move and include the sections of strength and limitations of study at the end of the discussion part.

Authors: Thank you for the recommendation. We agree that including the limitations of the study at the end of the discussion is the usual structure of manuscripts. But we believe it is better to discuss the limitations to the data we use for the empirical analysis in this study before we introduce the empirical strategy. This is because the discourse on the data limitations shape and connect to the empirical strategy we employ and elaborates the bounds within which the results should be interpreted. With the present structure, the reasons behind our non-conventional specification choices are also clear to the reader. We will request to be allowed to keep the limitations section at its current position within the manuscript.

Reviewer #1: I strongly recommend the authors seek English language revision for this manuscript. I believe this would help clarify some of the expressions and sentences that are currently not appropriate or incomprehensible.

Authors: Thank you for the recommendation. We have gone through the entire manuscript multiple times now. We have corrected grammatical errors and amended sentences to improve clarity. We also have had the manuscript professionally copy-edited.

Response to reviewer 2:

Reviewer #2: The manuscript is a very interesting read. A comprehensive analysis is done regarding mobility restrictions and way these are impacting spread of this disease. I believe that a fairly reasonable job is done regarding the estimation and analysis of very diversified data sets.

Authors: We thank the reviewer for the encouraging remarks.
